# The Role of Oxidative Stress in Ischaemic Stroke and the Influence of Gut Microbiota

**DOI:** 10.3390/antiox14050542

**Published:** 2025-04-30

**Authors:** Aleksandra Golenia, Piotr Olejnik

**Affiliations:** Department of Neurology, Medical University of Warsaw, 02-097 Warsaw, Poland; piotrek.olejnik2001@gmail.com

**Keywords:** oxidative stress, ischaemic stroke, gut microbiota, gut dysbiosis, neuroinflammation

## Abstract

Ischaemic stroke is the most prevalent stroke subtype, accounting for 80–90% of all cases worldwide, and remains a leading cause of morbidity and mortality. Its pathophysiology involves complex molecular cascades, with oxidative stress playing a central role. During cerebral ischaemia, reduced blood flow deprives neurons of essential oxygen and nutrients, triggering excitotoxicity, mitochondrial dysfunction, and excessive production of reactive oxygen and nitrogen species (RONS). Not only do these species damage cellular components, but they also activate inflammatory pathways, particularly those mediated by the transcription factor nuclear factor kappa-B (NF-κB). The pro-inflammatory milieu intensifies neuronal damage, compromises blood–brain barrier integrity, and exacerbates reperfusion-induced damage. Recent findings highlight the importance of the gut microbiota in modulating stroke outcomes, primarily through metabolic and immunological interactions along the gut–brain axis. Dysbiosis, characterised by reduced microbial diversity and an imbalance between beneficial and harmful strains, has been linked to increased systemic inflammation, oxidative stress, and worse prognoses. Specific gut-derived metabolites, including short-chain fatty acids (SCFAs) and trimethylamine N-oxide (TMAO), appear to either mitigate or intensify neuronal injury. SCFAs may strengthen the blood–brain barrier and temper inflammatory responses, whereas elevated TMAO levels may increase thrombotic risk. This narrative review consolidates both experimental and clinical data demonstrating the central role of oxidative stress in ischaemic stroke pathophysiology and explores the gut microbiota’s ability to modulate these damaging processes. Therapeutic strategies targeting oxidative pathways or rebalancing gut microbial composition, such as antioxidant supplementation, dietary modulation, probiotics, and faecal microbiota transplantation, present promising paradigms for stroke intervention. However, their widespread clinical implementation is hindered by a lack of large-scale, randomised trials. Future efforts should employ a multidisciplinary approach to elucidate the intricate mechanisms linking oxidative stress and gut dysbiosis to ischaemic stroke, thereby paving the way for novel, mechanism-based therapies for improved patient outcomes.

## 1. Introduction

Stroke is the second leading cause of death, the third leading cause of combined death and disability, and one of the main causes of adult dementia among non-communicable diseases worldwide [1,2]. Globally, over 12.2 million new strokes are reported each year, and around one in four adults over the age of 25 will suffer a stroke in their lifetime [1]. As the population ages, stroke prevalence is expected to increase. Of all strokes, ischaemic stroke is the most common type, accounting for 80–90% of cases, with the highest proportion occurring in high-income countries [1,2].

Ischaemic stroke occurs due to obstructed cerebral blood flow, leading to a reduction in oxygen, glucose, and nutrients critical for neuronal survival [3]. This results in a cascade of molecular, biochemical, and structural changes that underlie ischaemic stroke pathophysiology [3]. Several molecular mechanisms contribute to stroke pathology, including energy failure, loss of cellular ion homeostasis, acidosis, increased intracellular calcium levels, excitotoxicity, oxidative stress, arachidonic acid metabolite production, cytokine-mediated cytotoxicity, complement activation, blood–brain barrier (BBB) dysfunction, glial cell activation, and leukocyte infiltration [4,5]. While many of the above processes occur simultaneously, oxidative stress stands out as a major unifying mechanism that affects and exacerbates the others [6]. Thus, investigation of the molecular pathways of oxidative stress in ischaemic stroke is critical for developing effective therapies and improving patient outcomes [5,7].

Ischaemic stroke is characterised by many of the above-mentioned changes within the affected ischaemic core and the surrounding penumbra [3]. The main aim of ischaemic stroke treatment is to preserve the ischaemic penumbra, the area of hypoperfused tissue (reversible ischaemia) surrounding the infarct core (irreversible brain damage), by rapid restoration of blood flow [7,8]. Therefore, intravenous thrombolysis and/or endovascular therapy remain the gold standard treatment for patients with acute ischaemic stroke [8].

**Figure 1:**
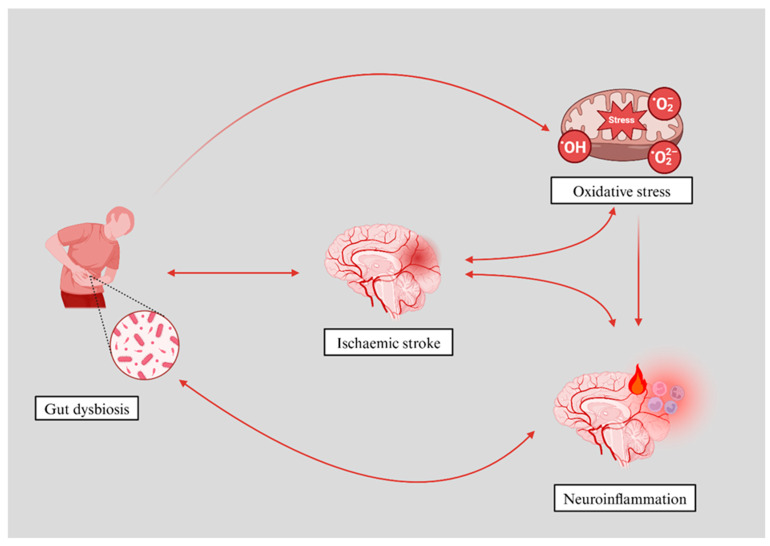
This figure illustrates the interplay between gut dysbiosis, oxidative stress, and neuroinflammation in the context of acute ischaemic stroke. Acute ischaemic stroke causes an oxidative burst, triggering an early inflammatory response and acute perturbations in gut microbiota. On the other hand, gut dysbiosis can exacerbate oxidative stress, which in turn contributes to the development and progression of ischaemic stroke. Also, oxidative stress directly influences the severity of neuroinflammation.

Oxidative stress results from an imbalance between reactive oxygen and nitrogen species (RONS) and the body’s antioxidant defences [9,10]. On the other hand, the gut microbiota, a key regulator of systemic inflammation, plays an integral role in stroke pathology [11]. Dysbiosis, or an imbalance in gut microbial composition, can exacerbate inflammatory responses and lead to a poorer patient outcome [12]. Studies have shown that the gut microbiota can influence neuroinflammation, which plays a critical role in brain recovery following an ischaemic stroke [11] (Figure 1).

This narrative review synthesises the current literature and provides a comprehensive overview of the evidence of the contribution of oxidative stress to ischaemic stroke pathophysiology, as well as the potential impact of gut microbiota on this process (Table 1).

## 2. Methods

To provide a comprehensive analysis of the topic, a detailed literature search was conducted using the PubMed and Google Scholar databases, covering the period up to 3 March 2025. The search included various types of studies, such as experimental and clinical research, as well as review papers. Specific search terms associated with the topic, including ‘short-chain fatty acids’, ‘oxidative stress’, ‘reactive oxygen species’, ‘reactive nitrogen species’, ‘antioxidants’, gut microbiota’, ‘gut microbiome’, and ‘acute ischaemic stroke’, were employed to filter titles and abstracts. To ensure thorough coverage of the topic, a manual review of reference lists was performed to expand the search. In addition, a search of ClinicalTrials.gov was conducted to identify ongoing but unpublished clinical trials. Articles that were not written in English or those that were not published as full scientific papers, such as conference abstracts or data sets, were excluded to maintain the relevance and quality of the review. The literature review was conducted independently by two investigators (A.G., P.O.) to reduce bias and ensure accurate data collection.

## 3. Oxidative Stress in Ischaemic Stroke

### 3.1. Pathophysiological Mechanisms Underlying Oxidative Stress

Free radicals are chemically reactive molecules containing one or more unpaired electrons, such as hydroxyl radical (^•^OH), superoxide (O_2_^•^⁻), or nitric oxide (^•^NO) [9,13]. Other molecules that can generate free radicals include hydrogen peroxide (H_2_O_2_) and peroxynitrite (ONOO⁻) [13]. Free radicals and related molecules are commonly referred to as RONS, and they include two categories of chemically reactive molecules: those containing oxygen, reactive oxygen species (ROS), and those containing nitrogen, reactive nitrogen species (RNS) [13,14,15]. ROS are produced as by-products of normal cellular processes, such as mitochondrial oxidative phosphorylation, where electron leakage during adenosine triphosphate (ATP) generation makes mitochondria a major site of ROS production [15]. However, other cellular sources significantly contribute to ROS production as well. For instance, red blood cells—despite lacking mitochondria—generate ROS mainly through the auto-oxidation of haemoglobin and the activity of NADPH oxidases [16]. These alternative sources are especially relevant in the context of acute stroke, where haemolysis and vascular dysfunction can amplify oxidative stress [16]. Moreover, a free radical can give rise to another secondary radical, e.g., superoxide (O_2_^•−^) can be converted into hydrogen peroxide (H_2_O_2_) and further into hydroxyl radical (^•^OH) [15]. Other sources of ROS include enzymatic activities such as nicotinamide adenine dinucleotide phosphate (NADPH) oxidase, which produces superoxide (O_2_^•−^); peroxisomes which, in turn, produce hydrogen peroxide (H_2_O_2_) during fatty acid oxidation; and xanthine oxidase, which produces ROS as a by-product during the conversion of hypoxanthine into uric acid [17,18]. At high concentrations, ROS may overwhelm the body’s antioxidant defence mechanisms and cause damage to cell structures, nucleic acids, lipids, and proteins [17]. At the same time, RNS are produced when nitric oxide (^•^NO) interacts with ROS or other reactive molecules, leading to the formation of peroxynitrite (ONOO^−^) [15]. RNS play a dual role in biological systems, participating in physiological signalling processes or causing pathological damage during oxidative stress [15]. Furthermore, at physiological concentrations, nitric oxide (^•^NO) is considered neuroprotective due to its role in vasodilation and neurotransmission [19]. However, under pathological conditions, free radicals may deplete nitric oxide (^•^NO) through the formation of peroxynitrite (ONOO^−^), thereby reducing the vascular bioavailability of nitric oxide (^•^NO) and leading to BBB dysfunction [19]. Similar to RNS, ROS also exhibit a dual role in biological systems. At physiological levels, ROS function as important signalling molecules involved in processes such as immune defence, cell proliferation, and redox signalling. However, excessive ROS accumulation disrupts redox homeostasis and leads to oxidative stress and cellular damage [17].

The body combats oxidative stress with both enzymatic and non-enzymatic antioxidant defences [17]. Enzymatic antioxidants include superoxide dismutase (SOD), which converts superoxide (O_2_^•−^) into hydrogen peroxide (H_2_O_2_); catalase, which converts hydrogen peroxide (H_2_O_2_) into water and oxygen; and glutathione peroxidase (GPx), which reduces hydrogen peroxide (H_2_O_2_) and lipid peroxides using glutathione [10,17]. Further, non-enzymatic antioxidant defence systems include some vitamins (Vitamin C, Vitamin E) and glutathione, a tripeptide that directly scavenges free radicals [10,17]. Oxidative stress can result from excessive ROS and/or RNS production and reduced antioxidant defence mechanisms [20,21]. When oxidative stress persists or overwhelms antioxidant defences, it can lead to cell death through both apoptosis and necrosis, as well as inflammation, aging, and various diseases such as atherosclerosis, cancer, diabetes, rheumatoid arthritis, post-ischaemic perfusion injury, myocardial infarction, cardiovascular diseases, chronic inflammation, stroke, septic shock, and other degenerative diseases in humans [9,10,17,20,21].

### 3.2. Oxidative Stress and Ischaemic Stroke: Advanced Mechanistic Insights

An ischaemic stroke occurs when there is a significant reduction in or complete blockage of cerebral blood flow to a specific region of the brain, depriving brain cells of the oxygen and glucose they need to function [22]. Further, oxidative stress plays a critical role in stroke pathophysiology because the brain is highly sensitive to damage caused by RONS [22]. First, the brain is particularly vulnerable to oxidative damage due to several factors, including high concentrations of peroxidisable lipids, low levels of protective antioxidants, high oxygen consumption, and elevated iron levels, which, in pathological conditions, act as pro-oxidants [22,23]. Second, oxidative reactions involving dopamine and glutamate also occur in the brain [22,24]. After an ischaemic stroke, a cascade of events is triggered in the brain due to reduced blood flow, oxygen deprivation, and subsequent cellular responses [23]. These mechanisms unfold in distinct phases, but begin rapidly with the onset of ischaemia [23]. Initially, an ischaemic stroke deprives neurons of oxygen and glucose, which are essential for ATP synthesis via oxidative phosphorylation [23]. Then, ATP depletion leads to the failure of energy-dependent ion pumps (e.g., Na^+^/K^+^-ATPase), resulting in ionic imbalances, including the influx of calcium (Ca^2+^), sodium (Na^+^), and chloride (Cl^−^) ions into the cell, and the efflux of potassium (K^+^) [23]. This causes depolarisation of the cell membrane, which subsequently leads to the development of cytotoxic oedema [23]. Furthermore, the massive release of glutamate activates ionotropic glutamate receptors, and prolonged receptor activation leads to excitotoxicity. This results in excessive calcium entry into neurons, triggering pathways that damage mitochondria and activate cellular enzymes such as proteases, lipases, and endonucleases [23]. Additionally, mitochondrial dysfunction and the activation of enzymes such as NADPH oxidase and xanthine oxidase produce ROS, including superoxide (O*_2_*^•−^) and hydrogen peroxide (H_2_O_2_). These ROS can attack cellular membranes, causing lipid peroxidation and the loss of membrane integrity, and also induce nucleic acid strand breaks and protein oxidation, impairing cellular functions and activating apoptotic pathways [23]. Finally, disturbances in calcium and glutamate homeostasis may also result in ischaemic necrosis or apoptosis [22,23].

**Figure 2:**
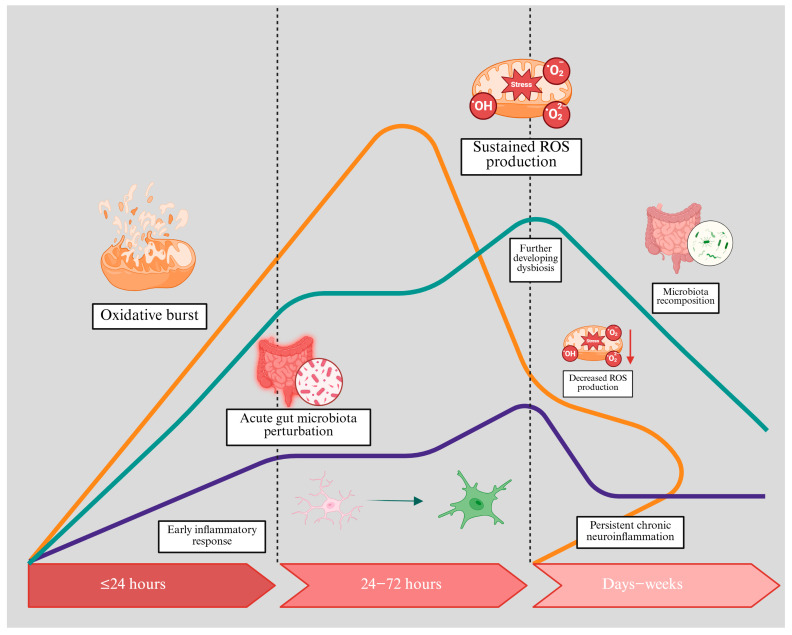
Temporal changes in oxidative stress levels (orange curve), neuroinflammation (blue curve), and gut microbiota (green curve) following acute ischaemic stroke. Within the first 24 h, an oxidative burst occurs, triggering an early inflammatory response and acute perturbations in gut microbiota. As the response extends from 24 to 72 h, sustained production of reactive oxygen species (ROS) exacerbates gut dysbiosis. Over the subsequent days to weeks, efforts at microbiota recomposition attempt to stabilise the gut environment and decrease ROS production. Despite these recovery mechanisms, persistent chronic neuroinflammation develops, highlighting potential intervention targets to prevent long-term damage.

Excitotoxicity and RONS activity induce neurons, oligodendrocytes, astrocytes, and microglia that initiate post-ischaemic inflammation [6,24]. RONS can activate transcription factors such as nuclear factor kappa-B (NF-κB), which upregulates pro-inflammatory cytokines (e.g., IL-1β, TNF-α, IL-6), chemokines, and adhesion molecules [25,26]. Endothelial cells and resident glial cells then amplify the inflammatory response by recruiting peripheral immune cells, which further increases oxidative stress through the release of more reactive species and proteolytic enzymes [27,28]. This vicious cycle exacerbates ischaemic damage by generating nitric oxide (^•^NO), ROS, and prostanoids, which can increase BBB permeability and cause secondary complications such as cerebral oedema and haemorrhagic transformation [29] (Figure 2).

### 3.3. Cerebral Ischaemia-Reperfusion Injury and ROS Generation

Cerebral ischaemia-reperfusion injury denotes tissue damage following blood flow restoration after a period of ischaemia or oxygen deprivation [30]. While restoration of blood flow is essential to prevent irreversible damage, reperfusion itself can paradoxically exacerbate tissue injury, further compromising organ function and viability [31]. Reperfusion injury involves multiple pathological processes, including oxidative stress, mitochondrial dysfunction, leukocyte infiltration, platelet activation and aggregation, complement activation, and BBB disruption [32]. These processes ultimately lead to cerebral oedema or haemorrhagic transformation [32].

The introduction of oxygen through restored blood flow to oxygen-deprived tissues leads to a significant increase in ROS production [33]. Excessive ROS not only directly damage all cellular components, including proteins, nucleic acids, and lipids, but also, if left unchecked, amplify pro-inflammatory molecular cascades and promote the recruitment and activation of additional leukocytes [23,32,33]. In an experimental study, increased ROS production was observed in the rat brain following cerebral ischaemia-reperfusion, as detected by electron spin resonance spectroscopy [34].

RONS production begins early in ischaemia and increases during reperfusion [35]. Mitochondria are one of the major sources of ROS after ischaemia-reperfusion [32]. It is well known that under ischaemic conditions, the electron transport chain in mitochondria is impaired, leading to the accumulation of succinate [36]. Upon reperfusion, the rapid oxidation of accumulated succinate by Complex II drives reverse electron transfer at Complex I, leading to excessive ROS production [37]. This process is a major contributor to oxidative damage during reperfusion [37]. Furthermore, the primary sources of free radicals during cerebral ischaemia-reperfusion include calcium-dependent oxidative enzymes such as xanthine oxidase, NADPH oxidase, and nitric oxide synthase, as well as the degradation of membrane and mitochondrial phospholipids [14]. The sudden increase in oxygen levels provides a substrate for several previously activated oxidative enzymes [14,35]. Upon reperfusion, xanthine oxidase catalyses the oxidation of hypoxanthine to uric acid, with superoxide (O_2_^•−^) and hydrogen peroxide (H_2_O_2_) being generated as by-products [14]. Moreover, the activation of NADPH oxidases contributes to elevated ROS production by transferring electrons from NADPH to molecular oxygen (O_2_), which generates superoxide (O_2_^•−^) and exacerbates neuronal injury [38]. Additionally, neuronal nitric oxide synthase (nNOS) produces nitric oxide (^•^NO) that can react with superoxide (O_2_^•−^) to form peroxynitrite (ONOO^−^), a potent oxidant that triggers nitrosative stress and further cell damage [14,19].

## 4. Gut Microbiota as a New Player in Ischaemic Stroke Pathophysiology

### 4.1. Overview of the Gut–Brain Axis in Ischaemic Stroke

The gut microbiota can be defined as the total composition of bacteria, archaea, and eukaryotes inhabiting the human gastrointestinal tract. With an estimated abundance of over 10^14^ microorganisms, the gut microbiota surpasses the number of all host cells tenfold [39]. The term gut microbiome, erroneously used interchangeably with gut microbiota, denotes by de facto the collective genomic composition of gut microorganisms [40]. A typical gut microbiota composition includes six phyla, such as Firmicutes, Bacteroidetes, Actinobacteria, Proteobacteria, Fusobacteria, and Verrucomicrobia. Among them, the Firmicutes and Bacteroidetes phyla are the most numerous, accounting for 90% of the gut microbiota [41,42]. The Firmicutes phylum consists of more than 200 genera, including *Lactobacillus*, *Bacillus*, *Clostridium*, *Enterococcus*, and *Ruminicoccus*, while the Bacteroidetes phylum consists mainly of the genera *Bacteroides* and *Prevotella* [42]. Under healthy conditions, the gut microbiota typically exhibits commensal properties that benefit the host. However, a state of microbial imbalance, known as dysbiosis, has been implicated in a wide range of human diseases [43]. Dysbiosis, in general, might be caused by decreased abundance of beneficial microorganisms, increased growth of harmful species, and an overall reduction in microbial diversity [43].

In recent years, research on the gut microbiota has focused not only on its contribution to host homeostasis, but also on its involvement in various diseases [41]. Moreover, studies have shown that the gut microbiota is involved in bidirectional gut–brain communication, referred to as the gut–brain axis [44,45]. The connection between the intestines and the central nervous system (CNS) involves multiple pathways, e.g., immune system regulation, the vagus and enteric nervous systems, neuroendocrine interactions, and the vascular system, all of which are influenced by the production and secretion of neuroactive compounds and metabolites [45,46]. Gut microbiota can communicate with the CNS mainly through neurotransmitters, including γ-aminobutyric acid (GABA), dopamine (DA), norepinephrine (NE), serotonin (5-HT), and histamine [47]. For instance, the *Bacteroides fragilis*, *Parabacteroides*, *Eubacterium*, and *Bifidobacterium* genera are recognised for their ability to synthesise GABA. Although GABA is unable to directly cross the BBB, it can affect the enteric nervous system and influence central nervous signalling via the vagus nerve [48]. Nevertheless, the gut microbiota may influence the production of neurotransmitters in the CNS by generating precursors that are capable of crossing the BBB [48].

The gut–brain axis has been implicated in the pathogenesis of neurological disorders such as multiple sclerosis and Alzheimer’s disease, as well as acute ischaemic stroke [49].

Certain microbiota-derived metabolites are particularly associated with ischaemic stroke, e.g., short-chain fatty acids (SCFAs) and trimethylamine N-oxide (TMAO) [49,50,51]. Trimethylamine, a precursor of TMAO, is produced by the gut microbiota from various dietary sources, including L-carnitine, phosphatidylcholine, choline betaine, and ergothioneine [52,53]. It is then absorbed in the small intestine and oxidised to TMAO in the liver by flavin-containing monooxygenase 3 [52]. TMAO has been demonstrated to be associated with ischaemic stroke incidents [53]. Furthermore, a meta-analysis by Farhangi et al. showed a positive dose-dependent association between TMAO concentration and the risk of stroke [52]. A potential explanation for this association is that TMAO increases platelet reactivity, thereby increasing the risk of thrombosis [54]. The gut microbiota may also be linked to ischaemic stroke through SCFAs, which are thought to have a protective effect against ischaemic stroke [55]. Generally, SCFAs, including acetate, propionate, and butyrate, are key metabolites produced in the colon through the bacterial breakdown of dietary fibre and are associated with gut–brain axis communication [56].

Since neuroinflammation is a critical factor in the progression of damage following ischaemia-reperfusion injury, the anti-inflammatory properties of SCFAs are considered to be particularly beneficial [55]. Nevertheless, the existing clinical data remain equivocal. For example, a study by Chou et al. in which 56 patients with acute ischaemic stroke underwent recanalisation therapy (30 with intravenous thrombolysis, 15 with mechanical thrombectomy, and 11 with both methods combined) revealed that the plasma levels of isovalerate, one of the SCFAs, were inversely correlated with stroke severity as measured by the National Institutes of Health Stroke Scale (NIHSS) both on admission and at discharge, while TMAO levels were not associated with stroke severity (as measured by the NIHSS) or functional outcome (as measured by the Modified Rankin Scale—mRS) [57]. Conversely, a study by Henry et al. involving 53 patients with acute ischaemic stroke who underwent mechanical thrombectomy, as well as 12 controls with stroke risk factors who underwent minimally invasive diagnostic angiography and elective neurointerventional procedures, found that plasma SCFA levels at the time of the stroke were not linked to stroke severity at presentation. Interestingly, higher SCFA levels at the time of the stroke were associated with elevated inflammatory markers [58]. Therefore, considering that both studies involved relatively small groups of subjects, the results should be interpreted with caution, and larger multi-centre studies are needed to confirm these findings.

### 4.2. Gut Microbiota and Ischaemic Stroke

Dysbiosis of the gut microbiota has been implicated in the development of various cardiovascular risk factors, including diabetes mellitus, hypertension, and obesity, which could subsequently lead to stroke. As a result, dysbiosis is also a common finding in patients with acute ischaemic stroke [12]. The majority of studies demonstrate that stroke patients exhibit reduced gut microbiome diversity [50]. Chang et al. conducted a study of 198 patients with acute ischaemic stroke due to small vessel occlusion, cardioembolism, or large artery atherosclerosis, and 200 healthy controls. The researchers isolated bacterial extracellular membrane vesicles from participant samples and extracted DNA from blood samples [59]. The study found a significant decrease in the Verrucomicrobia, Firmicutes, and Deferribacteres phyla, as well as significantly elevated levels of the Actinobacteria and Proteobacteria phyla [59]. Moreover, the study found that the Ruminococcaceae family and the Prevotella genus were significantly elevated in the poor outcome group (defined as those with an mRS score ≥ 3) compared to the good outcome group. Conversely, the Anaerococcus, Blautia, Dialister, Rothia, and Propionibacterium genera were significantly decreased in the poor outcome cohort [59]. It is particularly paradoxical that the Ruminococcaceae family and the Prevotella genus were increased in patients with a poor stroke outcome, as these bacteria produce SCFAs [60], which have anti-inflammatory properties that could potentially improve stroke outcomes [55]. Nonetheless, another study by Sun et al., which included 132 consecutive patients with acute ischaemic stroke due to anterior cerebral infarction, revealed that the abundance of SCFA-producing genera, including Bacteroides, Faecalibacterium, Roseburia, Ruminococcus, Coprococcus, and Butyricicoccus, was significantly decreased in the poor outcome cohort (defined as those with an mRS score ≥ 3). On the other hand, the pathogenic genus Enterococcus was enriched in this group [61]. These findings are consistent with the results of a study by Yashimiro et al. in which the researchers recruited 175 patients with acute ischaemic stroke within 24 h of symptom onset and 40 healthy controls [62]. The levels of acetic acid, the primary SCFA produced in the human body, were significantly lower in the ischaemic stroke patients compared to the healthy controls. Conversely, valeric acid levels were significantly elevated in the stroke cohort [62]. Moreover, the study found that valeric acid levels were positively correlated with high-sensitivity C-reactive protein levels and white blood cell counts, suggesting that gut dysbiosis in patients with acute ischaemic stroke is linked to host inflammation [62]. Furthermore, a study by Zeng et al. in aged C57BL/6J male mice demonstrated that valeric acid exacerbated neurological outcomes and intensified the inflammatory response, including increased blood IL-17 levels, following cerebral ischaemia [63]. Interestingly, the composition of the gut microbiota may also vary depending on the aetiology of ischaemic stroke. For instance, a study by He et al. found that the Bifidobacterium, Butyricimonas, Blautia, and Dorea genera, as well as the species Bifidobacterium longum, showed significant changes with high specificity in patients with large vessel occlusion stroke compared to those with cerebral small vessel disease and healthy individuals [64].

## 5. Mechanisms Linking the Gut Microbiota to Cerebral Ischaemia and Oxidative Stress

### 5.1. Gut Microbiota and Oxidative Stress

The gut microbiota is known to influence oxidative stress both directly and through various metabolites [65,66]. Interestingly, some probiotic organisms have the ability to produce their own antioxidants, such as SOD or catalase. Additionally, they can produce antioxidant metabolites such as folate and glutathione [67]. For instance, some bacteria from the *Lactobacilli* genera have the ability to synthesise glutathione at high levels and possess a fully functional glutathione system that includes glutathione peroxidase as well as glutathione reductase. These enzymes allow them to potentially influence the production of ROS [68]. Wanchao et al. have assessed the effects of inactivated *Lactobacillus* on cerebral ischaemia-reperfusion injury by using a middle cerebral artery occlusion and reperfusion model in male Sprague Dawley rats [69]. Their experiment showed that intravenous administration of inactivated *Lactobacillus* resulted in improved neurobehavioral scores and a significant reduction in infarct volume. Additionally, the study found that malondialdehyde levels (the marker of ROS and lipid peroxidation) decreased, while SOD activity increased in occipital lobe tissues, suggesting a reduction in oxidative stress levels [69]. Finally, not only the *Lactobacilli* genera, but also some species within the *Bifidobacterium* genera may reduce plasma levels of TMAO and both plasma and caecal levels of trimethylamine, potentially contributing to reduced oxidative stress [70]. However, there are too few studies assessing this association to draw definitive conclusions.

### 5.2. Metabolites Derived from Gut Microbiota and Oxidative Stress

Some metabolites produced by the gut microbiota [71], such as SCFAs, are thought to help alleviate the effects of oxidative stress [72]. Furthermore, SCFAs have the ability to improve BBB activity during acute ischaemic stroke [73]. According to an experimental study by Chen et al. involving aged male C57BL/6 mice (17–19 months), oral administration of butyrate decreased BBB permeability in a photothrombotic stroke model of focal cortical ischaemia [74]. Moreover, according to a small randomised, double-blind, cross-over study (ClinicalTrials.gov identifier: NCT00693355) involving 16 healthy volunteers, rectal administration of butyrate significantly increased glutathione concentration in colonic biopsies compared to placebo. Therefore, it may contribute to a reduction in oxidative stress levels [75]. These findings are consistent with the results of a study conducted by Wang et al. using mice with ischaemia-reperfusion injury induced by bilateral common carotid artery occlusion [76]. Oral administration of sodium butyrate exerts neuroprotective effects by mitigating oxidative stress, as evidenced by a significant increase in SOD activity and a significant decrease in malondialdehyde levels. Additionally, it reduces inflammatory responses, as indicated by significantly decreased levels of IL-1β, TNF-α, and IL-8 [76].

## 6. Clinical Implications of Targeting Gut–Brain Axis in Acute Ischaemic Stroke and Future Research Directions

Considering all of the above, targeting the gut microbiota may be a promising therapeutic strategy for both prevention and treatment of acute ischaemic stroke [77]. A Mendelian randomisation analysis by Qu et al. revealed that the genetically predicted enrichment of several SCFA-producing genera is causally associated with more favourable 90-day functional outcomes after ischaemic stroke. The authors suggested that SCFA-linked species may promote post-stroke recovery by mediating synaptic function [78].

Among the treatment options, dietary interventions, probiotics, and faecal microbiota transplantation are some noteworthy approaches [11,79]. A meta-analysis of 26 randomised controlled trials, collectively involving 2216 patients with stroke, reported that early enteral nutrition combined with probiotics significantly decreased the incidence of gastrointestinal complications and the incidence of infection, as well as shortening the length of hospital stay [80]. Complementing these findings, a retrospective study analysing *Bifidobacterium bifidum* supplementation in elderly patients with ischaemic stroke revealed significant decreases in their NIHSS scores over a 4-week follow-up period. In addition, inflammatory parameters, such as IL-6, IL-8, IL-1β, and TNF-α, were significantly decreased in the group receiving *Bifidobacterium bifidum* supplementation compared to the controls [81]. According to a systematic review by Savigamin et al., probiotics may potentially decrease neurological deficits following acute ischaemic stroke and reduce cerebral infarct volume due to their anti-inflammatory and antioxidant properties [82]. However, cross-study comparisons are hindered by the variability in probiotic bacterial strains used across research [82]. Additionally, certain probiotics have been shown to not only beneficially alter the gut microbiota composition, but also modulate neurotransmission systems [83,84]. For instance, Bercik et al. revealed that administration of *Bifidobacterium longum* NCC3001 in an animal model of chronic colitis normalised anxiety-like behaviours [84]. Similarly, Bravo et al. showed that treatment with *Lactobacillus rhamnosus* (JB-1) in healthy animals reduced stress-induced corticosterone levels and alleviated anxiety-like and depression-like behaviours, while also inducing region-specific changes in GABA receptor mRNA expression in the brain [83]. Notably, in both studies, these effects were absent in vagotomised animals, emphasizing the critical role of vagus nerve signalling at the level of the enteric nervous system in mediating observed behavioural changes [83,84]. Moreover, numerous animal-based studies have highlighted the role of faecal microbiota transplantation as a potential treatment for acute ischaemic stroke and its complications [85,86]. However, clinical data on this topic are still limited, making it difficult to draw definitive conclusions. There are currently no clinical trials registered on ClinicalTrials.gov evaluating the role of faecal microbiota transplantation in acute ischaemic stroke. Therefore, large, double-blind studies are needed to determine the causal effect of this procedure on stroke recovery.

## 7. Conclusions

Stroke remains a leading cause of mortality and long-term disability worldwide, with ischaemic stroke accounting for the majority of all stroke incidents. A growing body of evidence highlights the pivotal role of oxidative stress in the pathophysiology of acute ischaemic stroke, where excessive production of RONS precipitates neuronal damage and propagates inflammatory cascades. At the same time, the gut microbiota has emerged as a key modulator of stroke outcomes, influencing neuroinflammation and oxidative stress through multiple pathways and metabolites, including SCFAs and the modulation of neurotransmitter levels in both the enteric and central nervous systems. Dysbiosis, an imbalance in the gut microbial ecosystem, may exacerbate ischaemic damage by promoting inflammation and endothelial dysfunction, thereby broadening the scope of potential risk factors for acute ischaemic stroke.

Therapeutic strategies aimed at mitigating oxidative stress or restoring a healthy gut microbiota balance are promising new approaches for stroke treatment and prevention. Interventions such as the use of probiotics, targeted dietary interventions, or faecal microbiota transplantation may offer additional benefits when combined with established therapies such as thrombolysis and endovascular treatment. However, clinical data in this area remain limited. Large-scale, randomised controlled trials are needed to fully elucidate the therapeutic value of the modulation of gut microbiota composition in acute ischaemic stroke and to optimise interventions targeting oxidative stress. Fostering interdisciplinary research integrating neurology, microbiology, and immunology is crucial for the development of novel, mechanism-driven strategies to improve stroke outcomes.

## Figures and Tables

**Table 1:** A summary of the key concepts of ischaemic stroke pathophysiology, oxidative stress, and gut microbiota.

Topic	Key Points	Implications
Oxidative stress	Excess RONS produced by mitochondrial dysfunction, NADPH oxidase, and xanthine oxidase directly damage lipids, proteins, and nucleic acids, and activate inflammatory pathways.	Reducing oxidative stress may be a target for neuroprotective therapies.
Reperfusion injury	The sudden influx of oxygen intensifies RONS production and inflammation.	Interventions to modulate reperfusion-induced oxidative damage may improve functional outcomes.
Gut–brain axis	Dysbiosis can exacerbate oxidative stress and systemic inflammation.	Addressing gut dysbiosis may improve stroke outcomes and reduce post-stroke complications.
Gut microbiota metabolites	SCFAs (e.g., butyrate, acetate, propionate): generally neuroprotective, enhance BBB integrity, reduce inflammation. TMAO: implicated in increased thrombosis risk and unfavourable stroke outcomes.	Targeting these metabolites can modulate oxidative stress and inflammation. Potential for personalised nutrition/probiotics.
Therapeutic strategies	Antioxidants: counteract RONS (SOD mimetics, vitamin E). Probiotics/faecal microbiota transplantation: rebalance gut flora and potentially reduce oxidative stress and inflammation. Dietary interventions: high-fiber intake and specific prebiotic ingredients to foster SCFA production.	Adjunctive therapies can be combined with standard acute stroke care (e.g., thrombolysis, thrombectomy). Larger clinical trials are needed to confirm their efficacy.
Future directions	Larger-scale, randomised trials are needed to test microbiota-targeting and antioxidant therapies. Further research is required to elucidate the precise molecular mechanisms linking dysbiosis and oxidative stress. Exploring personalised medicine approaches could enhance treatment effectiveness.	A better understanding of the complex gut–brain oxidative pathways may lead to novel interventions that optimise stroke recovery.

RONS: reactive oxygen and nitrogen species, NADPH: nicotinamide adenine dinucleotide phosphate, SCFAs: short-chain fatty acids, TMAO: trimethylamine N-oxide, BBB: blood–brain barrier, SOD: superoxide dismutase.

## Data Availability

Not applicable.

## Abbreviations

The following abbreviations are used in this manuscript:

ATP	Adenosine triphosphate
BBB	Blood–brain barrier
Cl^−^	Chloride ion
CNS	Central nervous system
GPx	Glutathione peroxidase
H_2_O_2_	Hydrogen peroxide
IL-1β	Interleukin 1 beta
IL-6	Interleukin 6
IL-8	Interleukin 8
mRS	Modified Rankin Scale
Na^+^	Sodium ion
NADPH	Nicotinamide adenine dinucleotide phosphate
NF-κB	Nuclear factor kappa B
NIHSS	National Institutes of Health Stroke Scale
nNOS	Neuronal nitric oxide synthase
NO	Nitric oxide
O_2_^•−^	Superoxide anion radical
^•^OH	Hydroxyl radical
ONOO^−^	Peroxynitrite
RNS	Reactive nitrogen species
RONS	Reactive oxygen and nitrogen species
ROS	Reactive oxygen species
SCFAs	Short-chain fatty acids
SOD	Superoxide dismutase
TMAO	Trimethylamine N-oxide
TNF-α	Tumor necrosis factor alpha

## References

[1] Feigin V.L., Brainin M., Norrving B., Martins S.O., Pandian J., Lindsay P., Grupper M.F., Rautalin I. (2025). World Stroke Organization: Global Stroke Fact Sheet 2025. Int. J. Stroke.

[2] Feigin V.L., Abate M.D., Abate Y.H., Abd ElHafeez S., Abd-Allah F., Abdelalim A., Abdelkader A., Abdelmasseh M., Abd-Elsalam S., Abdi P. (2024). Global, Regional, and National Burden of Stroke and Its Risk Factors, 1990–2021: A Systematic Analysis for the Global Burden of Disease Study 2021. Lancet Neurol..

[3] Salaudeen M.A., Bello N., Danraka R.N., Ammani M.L. (2024). Understanding the Pathophysiology of Ischemic Stroke: The Basis of Current Therapies and Opportunity for New Ones. Biomolecules.

[4] Kuriakose D., Xiao Z. (2020). Pathophysiology and Treatment of Stroke: Present Status and Future Perspectives. Int. J. Mol. Sci..

[5] Woodruff T.M., Thundyil J., Tang S.-C., Sobey C.G., Taylor S.M., Arumugam T.V. (2011). Pathophysiology, Treatment, and Animal and Cellular Models of Human Ischemic Stroke. Mol. Neurodegener..

[6] Pawluk H., Tafelska-Kaczmarek A., Sopońska M., Porzych M., Modrzejewska M., Pawluk M., Kurhaluk N., Tkaczenko H., Kołodziejska R. (2024). The Influence of Oxidative Stress Markers in Patients with Ischemic Stroke. Biomolecules.

[7] Chamorro Á., Dirnagl U., Urra X., Planas A.M. (2016). Neuroprotection in Acute Stroke: Targeting Excitotoxicity, Oxidative and Nitrosative Stress, and Inflammation. Lancet Neurol..

[8] Patil S., Rossi R., Jabrah D., Doyle K. (2022). Detection, Diagnosis and Treatment of Acute Ischemic Stroke: Current and Future Perspectives. Front. Med. Technol..

[9] Uttara B., Singh A., Zamboni P., Mahajan R. (2009). Oxidative Stress and Neurodegenerative Diseases: A Review of Upstream and Downstream Antioxidant Therapeutic Options. Curr. Neuropharmacol..

[10] Golenia A., Leśkiewicz M., Regulska M., Budziszewska B., Szczęsny E., Jagiełła J., Wnuk M., Ostrowska M., Lasoń W., Basta-Kaim A. (2014). Catalase Activity in Blood Fractions of Patients with Sporadic ALS. Pharmacol. Rep..

[11] Benakis C., Liesz A. (2022). The Gut-Brain Axis in Ischemic Stroke: Its Relevance in Pathology and as a Therapeutic Target. Neurol. Res. Pract..

[12] Wang J., Zhang H., He J., Xiong X. (2022). The Role of the Gut Microbiota in the Development of Ischemic Stroke. Front. Immunol..

[13] Gilgun-Sherki Y., Melamed E., Offen D. (2001). Oxidative Stress Induced-Neurodegenerative Diseases: The Need for Antioxidants That Penetrate the Blood Brain Barrier. Neuropharmacology.

[14] Anaya-Fernández R., Anaya-Prado R., Anaya-Fernandez M.M., Guerrero-Palomera M.A., Garcia-Ramirez I.F., Gonzalez-Martinez D., Azcona-Ramirez C.C., Guerrero-Palomera C.S., Garcia-Perez C., Tenorio-Gonzalez B. (2024). Oxidative Stress in Cerebral Ischemia/Reperfusion Injury. OBM Neurobiol..

[15] Weidinger A., Kozlov A. (2015). Biological Activities of Reactive Oxygen and Nitrogen Species: Oxidative Stress versus Signal Transduction. Biomolecules.

[16] Daraghmeh D.N., Karaman R. (2024). The Redox Process in Red Blood Cells: Balancing Oxidants and Antioxidants. Antioxidants.

[17] Valko M., Leibfritz D., Moncol J., Cronin M.T.D., Mazur M., Telser J. (2007). Free Radicals and Antioxidants in Normal Physiological Functions and Human Disease. Int. J. Biochem. Cell Biol..

[18] Han D., Williams E., Cadenas E. (2001). Mitochondrial Respiratory Chain-Dependent Generation of Superoxide Anion and Its Release into the Intermembrane Space. Biochem. J..

[19] Reis P.A., de Albuquerque C.F.G., Maron-Gutierrez T., Silva A.R., Neto H.C.D.C.F. (2017). Role of Nitric Oxide Synthase in the Function of the Central Nervous System under Normal and Infectious Conditions. Nitric Oxide Synthase—Simple Enzyme-Complex Roles.

[20] Sies H. (2020). Oxidative Stress: Concept and Some Practical Aspects. Antioxidants.

[21] Sies H., Berndt C., Jones D.P. (2017). Oxidative Stress. Annu. Rev. Biochem..

[22] Allen C.L., Bayraktutan U. (2009). Oxidative Stress and Its Role in the Pathogenesis of Ischaemic Stroke. Int. J. Stroke.

[23] Saeed S.A., Shad K.F., Saleem T., Javed F., Khan M.U. (2007). Some New Prospects in the Understanding of the Molecular Basis of the Pathogenesis of Stroke. Exp. Brain Res..

[24] Ramiro L., Simats A., García-Berrocoso T., Montaner J. (2018). Inflammatory Molecules Might Become Both Biomarkers and Therapeutic Targets for Stroke Management. Ther. Adv. Neurol. Disord..

[25] Gloire G., Piette J. (2009). Redox Regulation of Nuclear Post-Translational Modifications During NF-ΚB Activation. Antioxid. Redox Signal.

[26] Morgan M.J., Liu Z. (2011). Crosstalk of Reactive Oxygen Species and NF-ΚB Signaling. Cell Res..

[27] Iadecola C., Anrather J. (2011). The Immunology of Stroke: From Mechanisms to Translation. Nat. Med..

[28] Jin R., Yang G., Li G. (2010). Inflammatory Mechanisms in Ischemic Stroke: Role of Inflammatory Cells. J. Leukoc. Biol..

[29] Moskowitz M.A., Lo E.H., Iadecola C. (2010). The Science of Stroke: Mechanisms in Search of Treatments. Neuron.

[30] Zhang M., Liu Q., Meng H., Duan H., Liu X., Wu J., Gao F., Wang S., Tan R., Yuan J. (2024). Ischemia-Reperfusion Injury: Molecular Mechanisms and Therapeutic Targets. Signal Transduct. Target. Ther..

[31] Anaya-Prado R., Toledo-Pereyra L.H., Lentsch A.B., Ward P.A. (2002). Ischemia/Reperfusion Injury. J. Surg. Res..

[32] Lin L., Wang X., Yu Z. (2016). Ischemia-Reperfusion Injury in the Brain: Mechanisms and Potential Therapeutic Strategies. Biochem. Pharmacol..

[33] Granger D.N., Kvietys P.R. (2015). Reperfusion Injury and Reactive Oxygen Species: The Evolution of a Concept. Redox Biol..

[34] Yamato M., Egashira T., Utsumi H. (2003). Application of in Vivo ESR Spectroscopy to Measurement of Cerebrovascular ROS Generation in Stroke. Free Radic. Biol. Med..

[35] Jung J.E., Kim G.S., Chen H., Maier C.M., Narasimhan P., Song Y.S., Niizuma K., Katsu M., Okami N., Yoshioka H. (2010). Reperfusion and Neurovascular Dysfunction in Stroke: From Basic Mechanisms to Potential Strategies for Neuroprotection. Mol. Neurobiol..

[36] Sanderson T.H., Reynolds C.A., Kumar R., Przyklenk K., Hüttemann M. (2013). Molecular Mechanisms of Ischemia–Reperfusion Injury in Brain: Pivotal Role of the Mitochondrial Membrane Potential in Reactive Oxygen Species Generation. Mol. Neurobiol..

[37] Tabata Fukushima C., Dancil I.-S., Clary H., Shah N., Nadtochiy S.M., Brookes P.S. (2024). Reactive Oxygen Species Generation by Reverse Electron Transfer at Mitochondrial Complex I Under Simulated Early Reperfusion Conditions. Redox Biol..

[38] Kleikers P.W.M., Wingler K., Hermans J.J.R., Diebold I., Altenhöfer S., Radermacher K.A., Janssen B., Görlach A., Schmidt H.H.H.W. (2012). NADPH Oxidases as a Source of Oxidative Stress and Molecular Target in Ischemia/Reperfusion Injury. J. Mol. Med..

[39] Thursby E., Juge N. (2017). Introduction to the Human Gut Microbiota. Biochem. J..

[40] Ursell L.K., Metcalf J.L., Parfrey L.W., Knight R. (2012). Defining the Human Microbiome. Nutr. Rev..

[41] Hou K., Wu Z.-X., Chen X.-Y., Wang J.-Q., Zhang D., Xiao C., Zhu D., Koya J.B., Wei L., Li J. (2022). Microbiota in Health and Diseases. Signal Transduct. Target. Ther..

[42] Rinninella E., Raoul P., Cintoni M., Franceschi F., Miggiano G., Gasbarrini A., Mele M. (2019). What Is the Healthy Gut Microbiota Composition? A Changing Ecosystem across Age, Environment, Diet, and Diseases. Microorganisms.

[43] DeGruttola A.K., Low D., Mizoguchi A., Mizoguchi E. (2016). Current Understanding of Dysbiosis in Disease in Human and Animal Models. Inflamm. Bowel Dis..

[44] Yamashiro K., Kurita N., Urabe T., Hattori N. (2021). Role of the Gut Microbiota in Stroke Pathogenesis and Potential Therapeutic Implications. Ann. Nutr. Metab..

[45] Kasarello K., Cudnoch-Jedrzejewska A., Czarzasta K. (2023). Communication of Gut Microbiota and Brain via Immune and Neuroendocrine Signaling. Front. Microbiol..

[46] Loh J.S., Mak W.Q., Tan L.K.S., Ng C.X., Chan H.H., Yeow S.H., Foo J.B., Ong Y.S., How C.W., Khaw K.Y. (2024). Microbiota–Gut–Brain Axis and Its Therapeutic Applications in Neurodegenerative Diseases. Signal Transduct. Target. Ther..

[47] Dicks L.M.T. (2022). Gut Bacteria and Neurotransmitters. Microorganisms.

[48] Pluta R., Januszewski S. (2022). Gut Microbiota Neurotransmitters: Influence on Risk and Outcome of Ischemic Stroke. Neural Regen. Res..

[49] Zhang S., Jin M., Ren J., Sun X., Zhang Z., Luo Y., Sun X. (2023). New Insight into Gut Microbiota and Their Metabolites in Ischemic Stroke: A Promising Therapeutic Target. Biomed. Pharmacother..

[50] Peh A., O’Donnell J.A., Broughton B.R.S., Marques F.Z. (2022). Gut Microbiota and Their Metabolites in Stroke: A Double-Edged Sword. Stroke.

[51] Chen G., Du X., Cui J., Song J., Xiong M., Zeng X., Yang H., Xu K. (2024). Role of Gut Microbiota in Ischemic Stroke: A Narrative Review of Human and Animal Studies. Neuroprotection.

[52] Farhangi M.A., Vajdi M., Asghari-Jafarabadi M. (2020). Gut Microbiota-Associated Metabolite Trimethylamine N-Oxide and the Risk of Stroke: A Systematic Review and Dose–Response Meta-Analysis. Nutr. J..

[53] Liu Y., Qu J., Xu J., Gu A., Deng D., Jia X., Wang B. (2023). Trimethylamine-N-Oxide: A Potential Biomarker and Therapeutic Target in Ischemic Stroke. Front. Neurol..

[54] Zhu W., Gregory J.C., Org E., Buffa J.A., Gupta N., Wang Z., Li L., Fu X., Wu Y., Mehrabian M. (2016). Gut Microbial Metabolite TMAO Enhances Platelet Hyperreactivity and Thrombosis Risk. Cell.

[55] Fang Z., Chen M., Qian J., Wang C., Zhang J. (2023). The Bridge Between Ischemic Stroke and Gut Microbes: Short-Chain Fatty Acids. Cell Mol. Neurobiol..

[56] Silva Y.P., Bernardi A., Frozza R.L. (2020). The Role of Short-Chain Fatty Acids From Gut Microbiota in Gut-Brain Communication. Front. Endocrinol..

[57] Chou P.-S., Yang I.-H., Kuo C.-M., Wu M.-N., Lin T.-C., Fong Y.-O., Juan C.-H., Lai C.-L. (2023). The Prognostic Biomarkers of Plasma Trimethylamine N-Oxide and Short-Chain Fatty Acids for Recanalization Therapy in Acute Ischemic Stroke. Int. J. Mol. Sci..

[58] Henry N., Frank J., McLouth C., Trout A.L., Morris A., Chen J., Stowe A.M., Fraser J.F., Pennypacker K. (2022). Short Chain Fatty Acids Taken at Time of Thrombectomy in Acute Ischemic Stroke Patients Are Independent of Stroke Severity But Associated With Inflammatory Markers and Worse Symptoms at Discharge. Front. Immunol..

[59] Chang Y., Woo H.G., Jeong J.H., Kim G.H., Park K.D., Song T.-J. (2021). Microbiota Dysbiosis and Functional Outcome in Acute Ischemic Stroke Patients. Sci. Rep..

[60] Fusco W., Lorenzo M.B., Cintoni M., Porcari S., Rinninella E., Kaitsas F., Lener E., Mele M.C., Gasbarrini A., Collado M.C. (2023). Short-Chain Fatty-Acid-Producing Bacteria: Key Components of the Human Gut Microbiota. Nutrients.

[61] Sun H., Gu M., Li Z., Chen X., Zhou J. (2022). Gut Microbiota Dysbiosis in Acute Ischemic Stroke Associated With 3-Month Unfavorable Outcome. Front. Neurol..

[62] Yamashiro K., Tanaka R., Urabe T., Ueno Y., Yamashiro Y., Nomoto K., Takahashi T., Tsuji H., Asahara T., Hattori N. (2017). Gut Dysbiosis Is Associated with Metabolism and Systemic Inflammation in Patients with Ischemic Stroke. PLoS ONE.

[63] Zeng X., Li J., Shan W., Lai Z., Zuo Z. (2023). Gut Microbiota of Old Mice Worsens Neurological Outcome after Brain Ischemia via Increased Valeric Acid and IL-17 in the Blood. Microbiome.

[64] He P., Jiang C., Ni J., Zhang X., Wu Z., Chen G., Huang J., Dai Z., Ji W., Li L. (2024). Identifying Gut Microbiota with High Specificity for Ischemic Stroke with Large Vessel Occlusion. Sci. Rep..

[65] Zhang J., Ling L., Xiang L., Li W., Bao P., Yue W. (2024). Role of the Gut Microbiota in Complications after Ischemic Stroke. Front. Cell Infect. Microbiol..

[66] Chen L., Wang X., Wang S., Liu W., Song Z., Liao H. (2025). The Impact of Gut Microbiota on the Occurrence, Treatment, and Prognosis of Ischemic Stroke. Neurobiol. Dis..

[67] Kunst C., Schmid S., Michalski M., Tümen D., Buttenschön J., Müller M., Gülow K. (2023). The Influence of Gut Microbiota on Oxidative Stress and the Immune System. Biomedicines.

[68] Feng T., Wang J. (2020). Oxidative Stress Tolerance and Antioxidant Capacity of Lactic Acid Bacteria as Probiotic: A Systematic Review. Gut Microbes.

[69] Wanchao S., Chen M., Zhiguo S., Futang X., Mengmeng S. (2018). Protective Effect and Mechanism of Lactobacillus on Cerebral Ischemia Reperfusion Injury in Rats. Braz. J. Med. Biol. Res..

[70] Wang Q., Guo M., Liu Y., Xu M., Shi L., Li X., Zhao J., Zhang H., Wang G., Chen W. (2022). Bifidobacterium Breve and Bifidobacterium Longum Attenuate Choline-Induced Plasma Trimethylamine N-Oxide Production by Modulating Gut Microbiota in Mice. Nutrients.

[71] Long J., Wang J., Li Y., Chen S. (2022). Gut Microbiota in Ischemic Stroke: Where We Stand and Challenges Ahead. Front. Nutr..

[72] Deng J., Li J., Li S., Zhang D., Bai X. (2025). Progress of Research on Short-Chain Fatty Acids, Metabolites of Gut Microbiota, and Acute Ischemic Stroke. Clin. Neurol. Neurosurg..

[73] Mathias K., Machado R.S., Stork S., Martins C.D., dos Santos D., Lippert F.W., Prophiro J.S., Petronilho F. (2024). Short-Chain Fatty Acid on Blood-Brain Barrier and Glial Function in Ischemic Stroke. Life Sci..

[74] Chen Z., Xin L., Yang L., Xu M., Li F., Zhou M., Yan T. (2023). Butyrate Promotes Post-Stroke Outcomes in Aged Mice via Interleukin-22. Exp. Neurol..

[75] Hamer H.M., Jonkers D.M.A.E., Bast A., Vanhoutvin S.A.L.W., Fischer M.A.J.G., Kodde A., Troost F.J., Venema K., Brummer R.-J.M. (2009). Butyrate Modulates Oxidative Stress in the Colonic Mucosa of Healthy Humans. Clin. Nutr..

[76] Wang R.-X., Li S., Sui X. (2019). Sodium Butyrate Relieves Cerebral Ischemia-Reperfusion Injury in Mice by Inhibiting JNK/STAT Pathway. Eur. Rev. Med. Pharmacol. Sci..

[77] Zhou S.-Y., Guo Z.-N., Yang Y., Qu Y., Jin H. (2023). Gut-Brain Axis: Mechanisms and Potential Therapeutic Strategies for Ischemic Stroke through Immune Functions. Front. Neurosci..

[78] Qu D., Jiang D., Xin Y., Yang G., Liang H., Wang L. (2024). Gut Microbiota and Functional Outcome after Ischemic Stroke: A Mendelian Randomization Study. Front. Immunol..

[79] Murthy P.M., CA J., Kandi V., Reddy M.K., Harikrishna G.V., Reddy K., JP R., Reddy A.N., Narang J. (2023). Connecting the Dots: The Interplay Between Stroke and the Gut-Brain Axis. Cureus.

[80] Chen X., Hu Y., Yuan X., Yang J., Li K. (2022). Effect of Early Enteral Nutrition Combined with Probiotics in Patients with Stroke: A Meta-Analysis of Randomized Controlled Trials. Eur. J. Clin. Nutr..

[81] Xin H., Zhang X., Li P., Li H., Feng G., Wang G. (2024). Bifidobacterium Bifidum Supplementation Improves Ischemic Stroke Outcomes in Elderly Patients: A Retrospective Study. Medicine.

[82] Savigamin C., Samuthpongtorn C., Mahakit N., Nopsopon T., Heath J., Pongpirul K. (2022). Probiotic as a Potential Gut Microbiome Modifier for Stroke Treatment: A Systematic Scoping Review of In Vitro and In Vivo Studies. Nutrients.

[83] Bravo J.A., Forsythe P., Chew M.V., Escaravage E., Savignac H.M., Dinan T.G., Bienenstock J., Cryan J.F. (2011). Ingestion of *Lactobacillus* Strain Regulates Emotional Behavior and Central GABA Receptor Expression in a Mouse via the Vagus Nerve. Proc. Natl. Acad. Sci. USA.

[84] Bercik P., Park A.J., Sinclair D., Khoshdel A., Lu J., Huang X., Deng Y., Blennerhassett P.A., Fahnestock M., Moine D. (2011). The Anxiolytic Effect of Bifidobacterium Longum NCC3001 Involves Vagal Pathways for Gut-Brain Communication. Neurogastroenterol. Motil..

[85] Pasokh A., Farzipour M., Mahmoudi J., Sadigh-Eteghad S. (2022). The Effect of Fecal Microbiota Transplantation on Stroke Outcomes: A Systematic Review. J. Stroke Cerebrovasc. Dis..

[86] Hediyal T.A., Vichitra C., Anand N., Bhaskaran M., Essa S.M., Kumar P., Qoronfleh M.W., Akbar M., Kaul-Ghanekar R., Mahalakshmi A.M. (2024). Protective Effects of Fecal Microbiota Transplantation against Ischemic Stroke and Other Neurological Disorders: An Update. Front. Immunol..

